# The Flavonoid Pathway in Tomato Seedlings: Transcript Abundance and the Modeling of Metabolite Dynamics

**DOI:** 10.1371/journal.pone.0068960

**Published:** 2013-07-26

**Authors:** Marian Groenenboom, Victoria Gomez-Roldan, Hans Stigter, Laura Astola, Raymond van Daelen, Jules Beekwilder, Arnaud Bovy, Robert Hall, Jaap Molenaar

**Affiliations:** 1 Biometris, Wageningen University and Research Center, Wageningen, The Netherlands; 2 Plant Research International, Wageningen, The Netherlands; 3 Netherlands Consortium for Systems Biology (NCSB), Amsterdam, The Netherlands; 4 Keygene N.V., Wageningen, The Netherlands; 5 Centre for BioSystems Genomics (CBSG), Wageningen, The Netherlands; Wake Forest University, United States of America

## Abstract

Flavonoids are secondary metabolites present in all terrestrial plants. The flavonoid pathway has been extensively studied, and many of the involved genes and metabolites have been described in the literature. Despite this extensive knowledge, the functioning of the pathway *in vivo* is still poorly understood. Here, we study the flavonoid pathway using both experiments and mathematical models. We measured flavonoid metabolite dynamics in two tissues, hypocotyls and cotyledons, during tomato seedling development. Interestingly, the same backbone of interactions leads to very different accumulation patterns in the different tissues. Initially, we developed a mathematical model with constant enzyme concentrations that described the metabolic networks separately in both tissues. This model was unable to fit the measured flavonoid dynamics in the hypocotyls, even if we allowed unrealistic parameter values. This suggested us to investigate the effect of transcript abundance on flavonoid accumulation. We found that the expression of candidate flavonoid genes varies considerably with time. Variation in transcript abundance results in enzymatic variation, which could have a large effect on metabolite accumulation. Candidate transcript abundance was included in the mathematical model as representative for enzyme concentration. We fitted the resulting model to the flavonoid dynamics in the cotyledons, and tested it by applying it to the data from hypocotyls. When transcript abundance is included, we are indeed able to explain flavonoid dynamics in both tissues. Importantly, this is possible under the biologically relevant restriction that the enzymatic properties estimated by the model are conserved between the tissues.

## Introduction

Flavonoids are a group of secondary metabolites widespread in the plant kingdom [1]. They are well known for their proposed health effects, and are abundant in, for example, fruits and tea [2], [3]. All terrestrial plants contain flavonoids, and they are present in all organs. Flavonoids have a large array of proposed functions, for example in flower pigmentation, development, pollination, and protection against UV radiation and pathogens [4]–[8].

**Figure 1:**
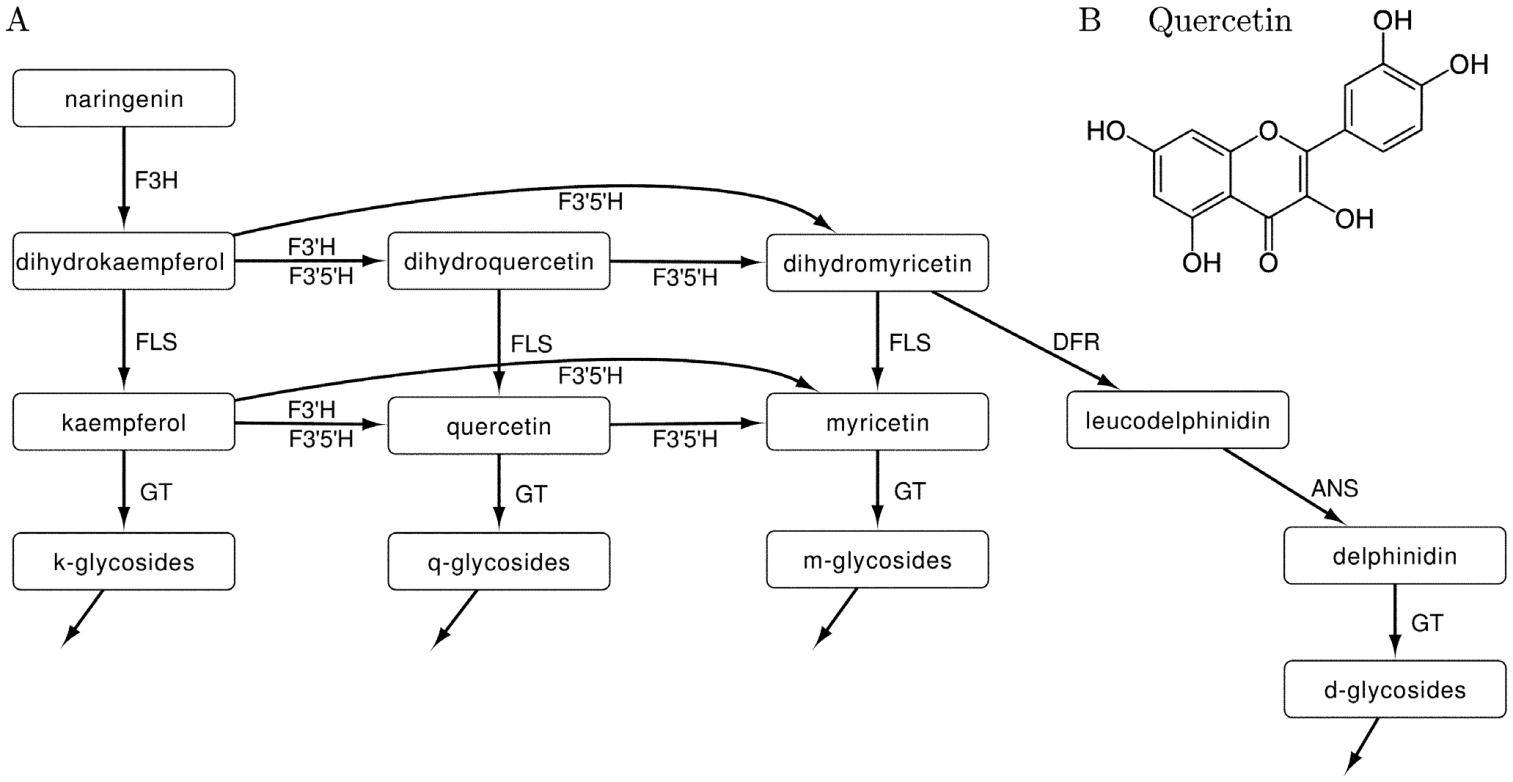
Synthesis and structure of flavonoids. (A) Scheme of the flavonoid pathway with the flavonoid aglycones at the nodes, and the enzymatic reactions on the edges. Nodes are labeled with the name of the metabolite and the corresponding variable in the model (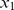 to 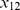). Edges are labeled with the enzyme name and the kinetic parameter estimated by the model (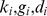). At the ends of the pathway the modified flavonols and anthocyanidins accumulate. Abbreviations: F3H, flavanone 3-hydroxylase; FLS, flavonol synthase; DFR, dihydroflavonol 4-reductase; ANS, anthocyanidin synthase. (B) Molecular structure of quercetin, one of the flavonoid aglycones.

Flavonoids are polyphenolic compounds (see Figure 1B), consisting of two aromatic rings with six carbon atoms (ring A and B) interconnected by a hetero cycle including three carbon atoms (ring C). They are classified into subgroups according to the structure and modifications of the central C-ring. The different groups of flavonoids typically accumulate after glycosylation, methylation or acetylation.

The flavonoid pathway has been extensively studied: over 6000 different flavonoids have been described, in many plant species the main enzymes have been identified and characterized, and transcript abundance, for example during fruit ripening, has been studied [9]–[12].

In tomato, many steps in the flavonoid pathway and the corresponding metabolites and enzymes have been identified [13]. The flavonoid pathway starts with the conversion of phenylalanine to 4-coumaroyl-CoA by phenylalanine ammonia-lyase (PAL), Cinnamate-4-Hydroxylase (C4H), and 4-coumaroyl:CoA-ligase (4CL). 4-coumaroyl-CoA can be converted into the first class of flavonoids, the chalcones, by the enzyme chalcone synthase (CHS). After modification of the chalcones by chalcone isomerase (CHI) the flavanones are formed, which in turn give rise to dihydroflavonols by flavanone 3*β* hydroxylase (F3H). At the level of the dihydroflavonols, the pathway branches into the two groups of end products. Flavonol synthase (FLS) synthesizes flavonols from dihydroflavonols, and dihydroflavonol 4-reductase (DFR), anthocyanidin synthase (ANS), and glycosyltransferases (GT) lead to the synthesis of anthocyanins. However, not only FLS and DFR are important for the branching into anthocyanins and flavonols. Anthocyanins found in tomato are derived from dihydromyricetin only, while it is suggested that the FLS has a strong preference for dihydrokaempferol and dihydroquercetin [14]. Hence, the branching of the pathway is regulated by the enzymes that function within the class of dihydroflavonols: flavonoid 3'-hydroxylase (F3'H) and flavonoid 3'5'-hydroxylase (F3'5'H); and by the expression of DFR and FLS (see Figure 1A).

In seedlings, the genes functioning in the biosynthesis of flavonoids are temporally regulated. Transcription and protein levels peak a few days after germination [15], [16]. CHS, CHI, F3H and FLS are generally classified as ‘early’ genes and DFR and ANS as ‘late’ genes.

Despite all available information on the separate steps in the pathway, there is still very little known about the behavior of the complete pathway *in vivo*. The extensive knowledge about the individual subnetworks makes the flavonoid pathway a good candidate for a systems biology approach in which experiments and modeling go hand in hand. The functioning of all the separate steps together in a pathway can be studied using mathematical or computational models. Relatively simple and concise models can already give important insights into a pathway and its efficacy. Until now, very few mathematical models for the flavonoid pathway have yet become available. Mathematical modeling was used very elegantly to unravel the kinetic mechanism of DFR in *Vitis vinifera* [17]. Another study explored properties of the network of flavonoid biosynthesis taken from the KEGG database [18]. A large data-based network has also been constructed for anthocyanin biosynthesis in *Arabidopsis thaliana* [19]. This network was used to search for the minimal set of metabolites and enzymes that lead to anthocyanin production. This minimal set differs per case, because different tissues and plants accumulate different flavonoids.

It is intriguing that one backbone of interactions together with a unique set of enzymes leads to the accumulation of different end products. In this paper we study the pathway leading to flavonol and anthocyanin accumulation in cotyledons and hypocotyls of tomato seedlings. We iteratively conducted experiments and adjusted the model to incorporate the information gained from the experiments. In this way we followed the so-called ‘experiment/modeling cycle’ several times [20]. We study the impact of changing enzyme concentrations on metabolite dynamics, and the usefulness of transcript abundance data in this matter.

## Results

### Measured Flavonoids

Tomato seedlings (*Solanum lycopersicum* L. cv. Moneymaker) were grown for 9 days and harvested on day 5 to 9 after sowing. To avoid fluctuations in flavonoid content due to circadian rhythm, seedlings were harvested each day at the same time. To study the differences between the different parts of the plants, the flavonoid content was measured separately in hypocotyls, cotyledons and roots using LC-MS. Concentrations were calculated from calibration curves of available standards (see Materials and Methods). In the roots flavonoid concentrations were were below the detection limit and for this reason only the data from the hypocotyls and cotyledons is used. Of the many possible flavonoids in the pathway shown in Figure 1A, only the methylated and/or glycosylated flavonols and anthocyanins (the filled circles) could be detected, meaning that all the other compounds are either absent or present below the detection limit. A list of measured compounds is shown in Table 1.

**Table 1:** Measured flavonoids in tomato seedlings during day 5 to 9 after sowing.

Kaempferol	-3-O-glucoside
	-3-O-rutinoside
	-3-O-diglucoside
	-3-O-rutinoside-7-O-glucoside
	-triglucoside
Quercetin	-3-O-glucoside
	-3-O-rutinoside
	-3-O-diglucoside
	-rutinoside-pentose
	-3-O-rutinoside-7-O-glucoside
	-triglucoside
	-3,7-O-diglucoside
	-3-O-diglucoside
Myricetin	-hexose
	-deoxyhexose-hexose
Laricitrin	–
	-hexose
Delphinidin	-hex-deoxyhex-p-coumaroyl-hex
Petunidin	-hex-deoxyhex-p-coumaroyl-hex
	-hexose-deoxy-feruloyl-hexose
Malvidin	-hex-deoxyhex-p-coumaroyl-hex

**Figure 2:**
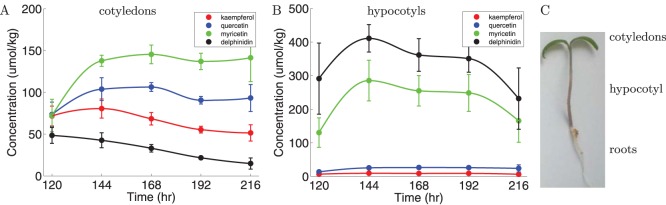
The concentration of flavonoids during seedling development. (A) measured flavonoids in cotyledons during seedling growth; (B) measured flavonoids in hypocotyls during seedling growth. Kaempferol in shown in green, quercetin in blue, myricetin in red, and anthocyanins in black. Shown are the sums of all glycosylated and methylated compounds derived from each type of aglycone. (C) Schematic of a seedling, showing the tissues used for the measurements: cotyledons and hypocotyls.

We sum all glycosylated and methylated compounds derived from each type of aglycone, and the resulting flavonol and anthocyanin concentrations are shown in Figure 2A and B. In the cotyledons mainly flavonols accumulate: myricitin is most abundant, kaempferol least abundant, and quercetin is present at intermediate levels. Although anthocyanins are present at low but detectable levels in the cotyledons, their concentrations decline during seedling growth. In the hypocotyls, myricetin and anthocyanins are present at high concentrations, whereas kaempferol and quercetin are present at low concentrations, compared to cotyledons. To interpolate the data, piecewise cubic Hermite interpolating polynomials (PCHIP) were fitted to the average measured concentrations.

### Mathematical Modeling of Metabolite Dynamics

The results of the experiments raises important question: What causes the differences between the accumulation patterns in cotyledons and hypocotyls? Is it necessary to consider different pathways in the two tissues, or different enzymatic rates?

To answer these questions we will investigate two mathematical models, which show different levels of refinement. First, we assume that all enzyme concentrations are constant over time. Because enzyme concentrations may vary between tissues, we allow them to differ between tissues. In a second approach, we take into account that enzyme concentrations may vary in time. Measured trends in transcript abundance are included in the model as approximations for enzyme concentrations. This extended model will be investigated in the next section.

In our models the interactions in the flavonoid pathway are described with ordinary differential equations (ODEs). With ODEs we can calculate the change in concentrations of the metabolites at each time point, depending on the production and conversion rates of the metabolites. The models describe the reactions starting with the synthesis of dihydrokaempferol by F3H and follow the scheme shown in Figure 1A down to the modified flavonols and anthocyanins. Each reaction from one metabolite to the next is facilitated by an enzyme. The flux involved in such a reaction depends on the concentration of the enzyme involved 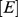, the rate constant 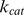 and saturation constant 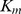 of that enzyme, and the concentration of the involved substrate 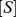. Since in our case the substrate concentration is low (under 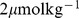), the rate of product formation 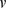 is given by the initial slope of the Michaelis Menten curve:
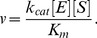
(1)


If 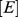, 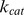, and 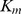 are known, the dynamics of the substrate concentrations (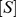) can be calculated from the model. In the literature, 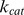 and 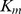 are available for some of the enzymes in the pathway. However, without knowledge of the enzyme concentrations in the seedlings the measured 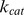 and 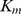 cannot be used to predict metabolite concentrations. We therefore estimate new parameter values that include both the enzymatic rates (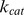 and 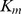) and the available enzyme concentration. Using the equations that describe the reactions leading from one metabolite to the next we estimate values for 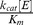 that result in the best fit to the observed metabolite data (Figure 2). The equations of the model can be found in the Materials and Methods section.

In addition to the production rates from one metabolite to the next, there is also a reduction rate for each end product. This is necessary to explain the observed decrease in the end products. This reduction rate can be contributed to degradation, export into other tissues or conversion of the compound to other products.

When the rates change, the time dependent metabolite concentrations predicted by the model change as well. By iteratively changing the rates, and comparing the model results with the data, we search for the best fit of the model to the data. To this end we use a genetic algorithm, that searches for a global optimum, followed by a local algorithm to improve on the solution (details can be found in the Materials and Methods section). The best fit is defined as having the smallest relative distance to the measured curves of the end products. Additionally, concentrations of intermediates should be below 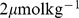, since they could not be detected.

**Figure 3:**
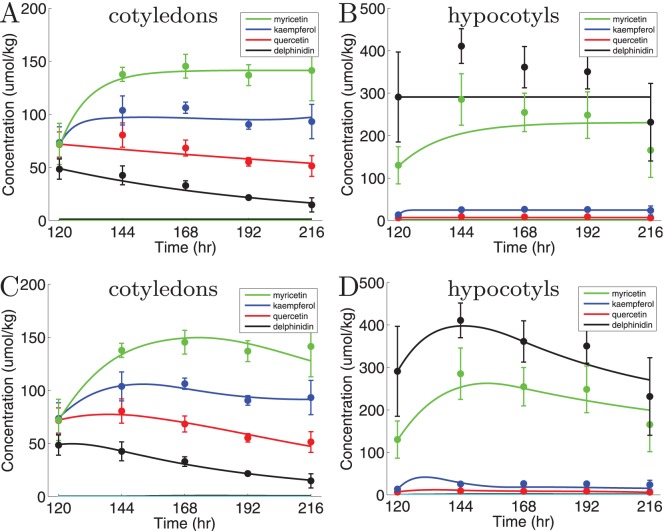
Fits obtained using the two models. Shown are the sums of all glycosylated and methylated compounds derived from each type of aglycone (filled circles with error bars) and the predicted curves by the model (lines). (A) and (B) results for the model using constant enzyme concentrations. The rates from one compound to the next are allowed to vary freely between the tissues. (C) and (D) results for the model using transcript abundance. The kinetic rates are constant between the tissues, except for the reduction rates of the anthocyanins and flavonol derivatives. Left graphs show cotyledons data and fits, right graphs hypocotyls data and fits. Kaempferol is shown in green, quercetin in blue, myricetin in red, and anthocyanins in black.

In Figures 3A and B we show results obtained with this model. All 22 rates were allowed to vary freely between the tissues. Despite this enormous freedom, the model is incapable of capturing the differences between the accumulation patterns in the two tissues. The data of the cotyledons can be fitted very well, but the fit to the data from hypocotyls is very poor. Especially the fast increase and decrease of myricetin and anthocyanins in hypocotyls are not yet captured (Figure 3B). What is correctly captured by this model is the low concentration of intermediate compounds (colored lines close to the 0 axis in Figure 3A and B), i.e. dihydrokaempferol, dihydroquercetin, dihydromyricetin, kaempferol aglycone, quercetin aglycone, myricetin aglycone, leucodelphinidin, delphinidin aglycone.

In conclusion, a model that uses constant rates does not explain the flavonoid dynamics in the seedlings satisfactorily.

### Mathematical Model that Combines Metabolic and Transcript Abundance Data

To improve the model discussed in the previous section, we here no longer assume that the rates (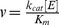) are constant over time, because 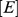 may be time dependent. *In vivo*, gene regulation changes enzyme concentration, which results in time dependent rates. In previous studies, basic regulatory processes were taken into account in, for example, flux balance analysis to improve model performance [21]. Instead of implementing largely unknown specifics of regulatory interactions in the flavonoid pathway, we will use transcript abundance data of the target enzymes as predictors for enzyme concentrations. Although many processes take place between transcript abundance and the resulting enzyme concentration, transcript abundance provides the model with information about the relative abundance of an enzyme in different tissues in the seedlings.

**Figure 4:**
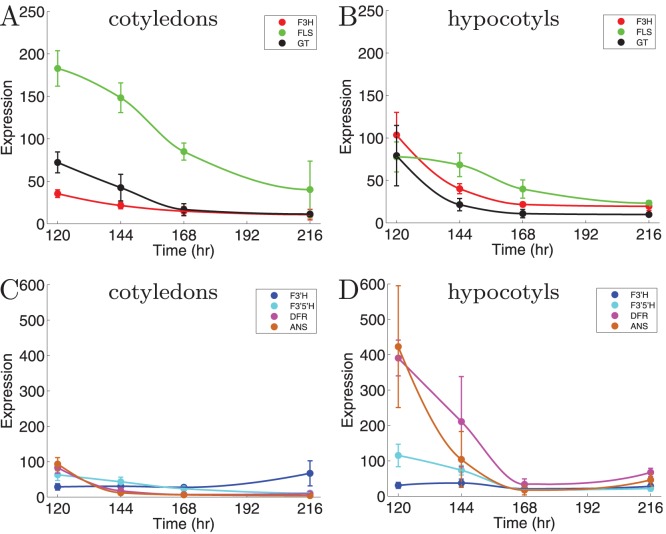
Relative transcript abundances(indicated as ‘expression’) of flavonoid genes during seedling development. Left graphs show cotyledons data, right graphs data from hypocotyls. (A) and (B) show the elative transcript abundances of F3H (red), FLS (green), and flavonoid 3-GT (black); (C) and (D) show the relative transcript abundances of F3'H (blue), F3'5'H (cyan), DFR (magenta), and ANS (orange).

Transcript abundance was quantified in tomato seedlings by using micro-array data, and values for relative expression of a subset of genes were confirmed by quantitative RT-PCR (Figure S1). The expression patterns of the flavonoid genes vary both over time and between the tissues. Almost all genes decrease greatly in expression during the experiment (see Figure 4). DFR and ANS are 4 times more highly expressed in the hypocotyls than in the cotyledons (Figures 4C and D). The expression of FLS in cotyledons is 2 times higher than in hypocotyls (Figures 4A and B).

The measured relative transcript abundance is included in the model. In the previous section we estimated the rate 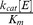 independently in both tissues, because enzyme concentration can vary between the tissues. Since we now include transcript abundance that was measured in the different tissues, we already have a measure for this variation of enzyme concentration between the tissues. For example, the production rate of kaempferol from dihydrokaempferol in cotyledons will decrease according to the FLS curve in Figure 4A, while the same reaction in hypocotyls will follow the FLS curve shown in Figure 4B. Remaining to estimate are the 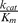 values. For simplicity, we assume that the 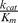 values do not change during seedling growth. These values are kinetic properties of the enzymes, and there is no reason to assume that these would differ between the tissues.

We therefore estimate values for these coefficients based solely on the data from the cotyledons and then apply the model with the estimated coefficients to the data from the hypocotyls. The differences between the tissues in the model are thus completely caused by the measured differences in transcript abundance. However, we do allow different degradation, or reduction rates of the end products, since it is possible that these also differ between the tissues, and the identity of the enzymes involved in that step is not known.

Using this approach we obtained the results shown in Figures 3C and D. This model correctly fits the cotyledons data. When the model and estimated parameter values are tested on data from hypocotyls, we find that this model can also explain the dynamics in the hypocotyls. From these fits we conclude that our eventual model explains the large difference in flavonol and anthocyanin accumulation between the cotyledons and the hypocotyls very well. In particular, the model explains the strong increase in myricetin and anthocyanins in the hypocotyls, which was not possible without incorporating the transcript abundance data. This result implies that the time dependent trends of the genes are able to explain the dynamics of the metabolite concentrations; and that the differences in transcript abundances measured in the two tissues turns out to be sufficient to explain the different metabolite accumulation patterns.

All rates from one metabolite to the next vary between the tissues according to the measured transcript abundance curves. That results in a higher flux towards myricetin and the anthocyanins in the hypocotyls, while the estimated reaction rate remains constant. Only the reduction rates are estimated separately in the two tissues. We find that the reduction rates are all estimated within the same order of magnitude. Degradation of kaempferol and quercetin is estimated to be 2 to 4 times higher in hypocotyls than in cotyledons. Anthocyanin degradation is up to 5 times higher in cotyledons, degradation of myricetin is similar in both tissues.

Due to the network topology there are multiple routes to end products. The combination of this network topology and the lack of information on intermediate dynamics, leads to identifiability problems. This means that multiple sets of rates fit the data more or less equally well. To study this problem in more detail 250 independent runs were executed to fit the data, and we found that all lead to a good fit to both cotyledons and hypocotyls. The average fit has a total sum of squares of 2.18, the best fit has a value of 0.78, the worst 3.87. Shown in Figure 3C and D is a fit of 1.89.

We found that in many estimated parameter sets not all connections are used (reaction rate is zero). For example, myricetin is in most fits not produced by FLS. Generally, more edges in a network will lead to a better fit. Here this is not the case, because the time dependent trends of transcript abundance on the edges might not result in the correct metabolite dynamics. Only 5.2% of the estimated parameter sets use all 22 reactions, all the other solutions exclude 1 to 5 edges. Some connections are used in all fits, and are therefore indispensable for correct metabolite accumulation.

**Figure 5:**
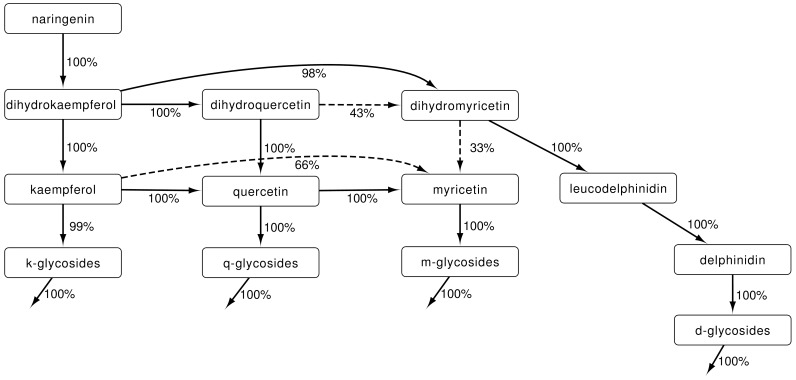
Summary of 250 independent parameter estimations. Shown is the modeled flavonoid network leading to the accumulated end-products: keampferol-, quercetin-, myricetin- and delphinidin-glycosides. Edges are shown with a solid line if they occur in more than 2/3 of the 250 independent parameter estimations, they are dashed when the occur less often. Shown next to each edge are the facilitating enzyme and the percentage of runs that used this edge. The double edges by F3'H and F3'5'H from 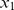 to 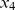 and from 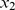 to 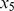 are never zero at the same time, that is, there is always a connection between 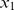 and 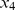, and between 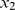 and 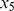. Arrows without endpoints represent reduction rates due to, e.g., degradation.

In the scheme shown in Figure 5 the results of the 250 runs are summarized. The edges are labeled with the percentage of simulations that the edge occurred. We can now infer the routes through the pathway that lead to the different end products. Logically, kaempferol is produced via dihydrokaempferol and FLS, since there is no other route. Quercetin can be produced via two different routes. We always find a connection of dihydrokaempferol to dihydroquercetin, and from kaempferol to quercetin. This reaction can be either facilitated by F3'H or F3'5'H, or both. There is also always a connection from dihydroquercetin to quercetin. These findings indicate that quercetin is produced through both routes: via F3'H or F3'5'H and kaempferol, and via FLS and dihydroquercetin. We find that myricetin is in all 250 cases produced from quercetin, while in only 33% of the cases it is produced from dihydromyricetin as well. These results suggest that FLS activity on dihydromyricetin is not necessary to explain the dynamics. This finding is in agreement with the hypothesis by Bovy et.al [14] that FLS has a low affinity for dihydromyricetin. The most interesting enzyme in this part of the pathway is F3'5'H. It could facilitate many reactions and it is crucial in the production of dihydromyricetin, and consequently indispensable for anthocyanin production. We find that it should facilitate almost all reactions it was implicated in to result in correct metabolite accumulation (Figure 5). Only the reaction from dihydroquercetin to dihydromyricetin is not necessary when it is possible that dihydromyricetin is directly produced from dihydrokaempferol.

**Figure 6:**
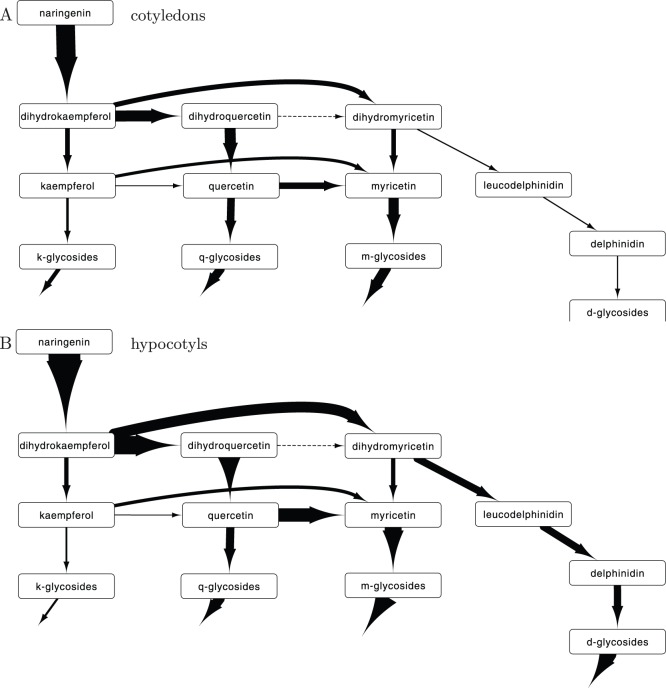
Accumulated flux of metabolites predicted by the model using transcript abundances. Shown is the the total flux of product throughout day 5 to 9, which is expressed in *µ*mol kg^−1^. Arrow thickness increases for increasing accumulated flux. The parameters of Figure 3 were used to calculate accumulated flux. (A) Fluxes in cotyledons and (B) in hypocotyl. Arrows without endpoints represent reduction rates due to, e.g., degradation.

It is difficult to infer from the model topology and the transcript abundance exactly how the full model is able to explain the difference in flavonoid accumulation in the two tissues. This however, is clear when looking at the flow of compounds through the network. To this end we calculate the total flux from compound to compound. We multiply the transcript abundance with the catalytic rate and the concentration of the compound, and sum this over time. The resulting accumulated flux is shown in Figure 6. The arrow thickness represents accumulated flux in the network.

The largest differences in flavonoid accumulation between the two tissues lies in the larger accumulation of myricetin and delphinidin derivatives in the hypocotyls. From the calculated fluxes can be seen that the flux from dihydrokaempferol towards dihydroquercetin and dihydromyricetin is much larger in hypocotyls than in the cotyledons (Figure 6). In the hypocotyls there is a much larger influx into the pathway, caused by increased expression of F3H. The combination of reduced expression of FLS and enlarged expression of F3'5'H forces this flux towards dihydroquercetin and myricetin. From dihydromyricetin it continues towards delphinidin, and from dihydroquercetin the flux can only continue towards quercetin. Again the increased expression of F3'5'H drives the flux towards myricetin. Please note that the differences in fluxes is solely caused by the measured transcript abundance. The accumulated fluxes shown in Figure 6 are derived from only one possible solution, the one that is also used for Figure 4. When other solutions are used, the accumulated fluxes can differ because the lack of some connections will lead to an increase in flux through other connections (see Figure 5). However, all solutions show an increased flux into the hypocotyl network, and increased flux towards dihydromyricetin and myricetin. Concluding, the different expression of genes in the pathway leads to distinctive patterns in flux towards certain compounds, leading to the observed accumulation patterns.

## Discussion

Despite the detailed information available about enzymes and metabolites, the functioning of the flavonoid metabolic pathway *in vivo* is still poorly understood [22]. Here we combined experiments with mathematical modeling to unravel the pathway leading to flavonol and anthocyanin accumulation in tomato seedlings.

Four types of flavonoids accumulate in tomato seedlings: kaempferol, quercetin, myricetin, and delphinidin derivatives. For each of these four end products, concentrations were measured on day 5 to 9 in cotyledons and hypocotyls of tomato seedlings. Interestingly, the accumulation patterns vary between the tissues, although they share the same backbone of interactions (Figure 1A). Questions that arise are: what causes the differences between the tissues, and do we have to consider different routes or networks to explain these differences?

We found that a mathematical model with constant rates from and to metabolites was unable to fit the data, even though all parameters were allowed to vary between the tissues. The fit to cotyledons data was reasonable, but the fit to data from hypocotyls was poor.

Expression patterns of genes coding for known flavonoid enzymes were measured and incorporated in the model. The extended model then allowed for a good fit to the data. In the model, the only difference between the tissues is in the measured transcript abundance patterns, whereas the rates at which the enzymes convert substrates are taken to be the same for both tissues. Incorporation of enzyme dynamics is still not often used in metabolic modeling, but turns out to be essential in the present system. We found that transcript abundance data is very useful in explaining the observations, even though there are many processes taking place between transcript abundance and enzyme concentration, which we could not take into account. In conclusion, we have shown that the used transcript abundance patterns are useful data to estimate enzyme concentrations, and that the quantitative information in relative transcript abundance was able to explain the difference between flavonoid accumulation in cotyledons and hypocotyls.

In preliminary studies, we have considered some other topologies. We first attempted to fit the data with a more concise model that did not include F3'H and F3'5'H. That model described the reactions between groups of flavonoids: from flavanones to dihydroflavonols (F3H), and from dihydroflavonols to anthocyanins (DFR, ANS and GT) or flavonols (FLS and GT). Even though transcript abundance data was used, we were unable to find rates that could explain the data in both cotyledons and hypocotyls. Another topology included only kaempferol, quercetin, and myricetin, including dynamics of FLS, F3'H, F3'5'H, and GT. This model also failed to fit the data. The only topology that currently can explain the data is the topology shown in Figure 1A. Our results suggest that F3'5'H directed flux towards dihydromyricetin and myricetin at both the level of the dihydroflavonols and the flavonols is crucial for explaining flavonoid accumulation in the seedlings.

Although the current pathway with multiple routes to the different end products results in very good fits, this topology leads to identifiability problems. The model described here is a typical “sloppy model”: almost all parameters have a sloppy spectrum of parameter sensitivities [23]. Unfortunately, the knowledge that intermediates should be below detection level is not specific enough to give quantitative predictions. Additional information about the concentration of intermediates may allow for estimation of these fluxes. Since such detailed measurements are technically limited, our future research will focus on unraveling the network further using the current model and qualitative biological knowledge on the enzymes in combination with parameter reduction [24]. Another important topic in the functioning of the flavonoid pathway is the impact of the observed interactions between enzymes. Recently it has been shown that FLS and DFR are able to directly associate with CHS [25]. More mathematical modeling is needed to explore the effect of these complexes on flavonoid accumulation.

The sloppiness of the model does not prohibit biologically relevant conclusions to be drawn. In our case we can use the model to study through what routes the end products are most likely produced. We found that the connection of dihydromyricetin to myricetin is dispensable for a good fit to the data. This is in accordance with the previous hypothesis that FLS might not use dihydromyricetin as a substrate [14]. It could be the case that ANS rather than FLS, mediates synthesis of myricetin, because it is known that ANS is capable of acting as an FLS [26].

The model described here can be used to test candidate genes. For example, many glycosyltransferases have been putatively annotated (in total over 200 in the tomato genome), but their specificity remains unknown. Mathematical modeling approaches to link these transferases to substrates is currently being studied as a valuable predictor tool for gene-function analysis [27], [28].

## Materials and Methods

### Plant Material

Sterilized seeds of tomato (*Solanum lycopersicum* L. cv. Moneymaker) were sown in pots filled with 70 ml of half Hoagland nutrition solution/0.5% Agar. Pots were placed in a growth chamber under a cycle of 16 h light (100 *µ*mol m
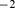
s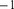) and 8 h of darkness at 25°C (standard growth-light conditions). Samples were harvested each day at 13h00 from day 5 to 9 after sowing. After harvesting we divided the seedlings into cotyledons, hypocotyls, and roots; ground with liquid nitrogen; freeze-dried and stored at -80°C. For each day three biological replicates were harvested (3 pots per day).

### Flavonoid Detection and Quantification

For flavonoid detection 5–10 mg dry weight (DW) of seedling samples (cotyledons and hypocotyls) were extracted with 1∶70 (v/v) volume of water/methanol (70%) solution acidified with 1% (v/v) formic acid. Extracts were sonicated for 15 min, centrifuged and filtered through a 0.45 *µ*m inorganic membrane filter (Sarturius stedim, Biotech). The resulting extract was used for LC-PDA-QTOF MS analysis. Chromatography separation was performed using a Luna C18 (2) precolumn (2.0 x 4 mm) and an analytical column (2.0 x 150 mm, 100, particle size 3 um) from Phenomenex. The injection volume of standards and samples was 5 *µ*l. The mobile phase consisted of water/formic acid (1000∶1, v/v; eluent A) and acetonitrile/formic acid (1000∶1, v/v; eluent B) with flow set at 0.19 mL.min^−1^. The gradient elution program was the following: 5% B to 35% B in 45 min, after which the column was washed for 15 min and equilibrated before the next injection. The column temperature was maintained at 40°C and the samples at 20°C. After chromatography, the UV absorbance of the column eluent was measured using a Waters 2996 PDA (range from 240 to 600 nm) and ESI-MS analysis was performed using a QTOF Ultima V4.00.00 mass spectrometer (Waters- Corporation, MS technologies) in negative mode. Collision energy of 10 eV was used for full-scan LC-MS in the m/z range 100 to 1,500. Leucine enkephalin, [M H]  = 554.2620, was used for online mass calibration (lock mass). Acquisition and visualization of the LC-PDA-QTOF data was performed using MassLynx 4.0 software (Waters). Identification of the different flavonoids detected in cotyledons and hypocotyls samples was based on the accurate mass and retention time previously determined in the literature. To determine the concentration of flavonols (quercetin, kaempferol and myricetin) and anthocyanin derivatives, calibration curves were performed with the available commercial standards. Standard stock solutions were prepared in methanol (1%v/v Formic Acid) at different concentrations: 0.2, 0.5, 1, 2 and 10 *µ*g/ml, and UV and MS signals were used to calculate the linear regression of the signal with respect to concentration. In case of metabolites for which no standards were available, the UV calibration curve of the most related compound was used. In these cases the molar absorption constant of the compound was assumed to be the same for the available compound and the derivatives. Quercetin 3-O-rutinoside (Sigma) calibration curve was used for quercetin derivatives, kaempferol 3-O-rutinoside (Sigma) for kaempferol derivatives, myricetin 3-O-glucoside (Extrasynthese) for myricetin derivatives and delphinidin 3-glucoside (Extrasynthese) for all the anthocyanins detected in the tomato seedlings [29].

### Transcript Abundance Analysis and Gene Selection

Transcript abundance data of the different flavonoid related genes were obtained from a transcriptomic analysis performed with the same plant material that was used for the flavonoid detection [29]. In brief, RNA was isolated from the three biological replicates of 4 different days (5, 6, 7 and 9) and 2 tissues (hypocotyls and cotyledons) using QuickGene^TM^ RNA Cultured Cell HC Kit (Fujifilm, USA). Transcript abundance analysis was performed using a customer array from the EUSOL project. The Affymetrix EUTOM3 GeneChip was analysed according to the manufacturer's instructions. EUTOM3 is a cDNA microarray representing ca. 32,000 genes representative of the tomato genome. The preprocessing and normalization of all CEL files was performed with RMA algorithm [30], [31]. The EUTOM3 Affymetrix microarrays were annotated with the official annotation for the tomato genome provided by the International Tomato Annotation Group (ITAG).

Candidate flavonoid genes were selected based on prior biological knowledge (see Table 2). Genes encoding the enzymes of the flavonoid pathway have largely been identified. However, the tomato genome is complex, in the sense that often more than one copy of a gene is present [32]. For all enzymes, a single candidate with high homology to a well-characterized protein from Petunia x hybrida (a closely related plant) could be identified among the annotated genes (Table S1). In the case of GT, enzymes encoded by multiple genes could facilitate the same step in the pathway. In this study however, we used only genes that were previously described or were similar to previously described genes and we did not test on enzyme synthesis expression levels due to other genes than the ones that were selected. Quantitative RT PCR experiments were performed on the same RNA that was used for microarray experiments. 1 *µ*g of total RNA was used for cDNA synthesis using the iScript cDNA Synthesis Kit (Bio-Rad). Realtime PCR reactions were carried out in triplicate in a total volume of 20 ul containing 10 *µ*l of 2× iQ SYBR Green Supermix (Bio-Rad), 0.3 *µ*M of forward and reverse primer and 10 ng of cDNA in a MyiQ real-Time PCR machine from Bio-Rad. The following PCR program was used: 95°C for 3 min, followed by 40 cycles of 95°C for 15 sec and 60°C for 1 min. Ribosomal protein L33 was used as a reference gene. Relative transcript abundance was calculated as: 2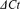, where 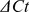 = Ct CnVS Ct L33. Oligonucleotides used are shown in Table 3.

**Table 2:** Selected genes from tomato seedlings.

function	id number	*A. thal.*	ref
F3H	Solyc02g083860	at3g51240.1	[14]
F3'H	Solyc03g115220	at5g07990.1	[14]
F3'5'H	Solyc11g066580	at5g07990.1	[33]
FLS	Solyc11g013110	at5g08640.2	[14]
DFR	Solyc02g085020	at5g42800.1	[33]
ANS	Solyc08g080040	at4g22880.2	[33]
GT	Solyc10g083440	at5g17050.1	[14]

**Table 3:** Oligonucleotides used for qRT-PCR.

Name	locus	Forward primer	Reverse primer
[rgb]0.8,0.8,0.8 L33	Solyc03g096360	GGGAAGAGGCTGGGATACATC	AGGAGGCAAATTGGACTTGAAC
FLS	Solyc11g013110	GAGCATGAAGTTGGGCCAAT	TGGTGGGTTGGCTCATTAA
[rgb]0.8,0.8,0.8 DFR	Solyc02g085020	TCCGAAGACGACAACGGTTT	TGACAAGCCAAGAGCCGATAA
F35H	Solyc11g066580	GGCAATTGGACGAGATCCTG	AAGGAACCTCTCGGGAGTGAA

The obtained transcript abundance curves are shown in Figure 4. As transcript abundance values the RMA (Robust Multichip Average) values were used, which were generated from Affymetrix output data by a background adjustment and a quantile normalization, according to standard procedures [30]. Smooth curves were fitted to the measured transcript abundance with a PCHIP (Piecewise Cubic Hermite Interpolating Polynomial). The obtained functions describe relative changes in enzyme expression and were used in the mathematical model.

### Computational Methods

The reactions of the flavonoid pathway (Figure 1A) are described using ordinary differential equations:
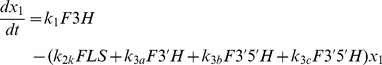
(2)

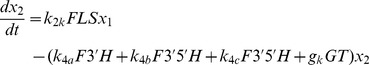
(3)

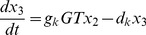
(4)

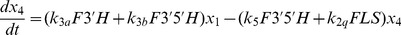
(5)

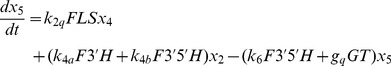
(6)

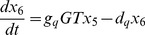
(7)

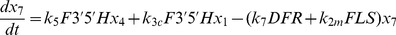
(8)

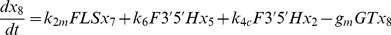
(9)

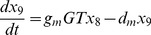
(10)

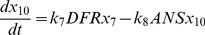
(11)

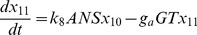
(12)

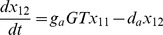
(13)


Here, 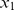 to 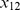 represent the metabolite concentrations as shown in Figure 1A. 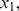 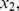 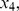 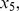 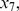 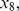 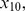 and 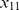 represent aglycone flavonoids, 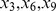 and 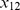 modified (methylated or glycosylated) flavonoids. 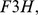 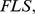 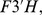 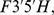 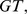 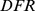 and 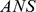 represent the enzyme concentrations; 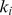 the enzymatic rates; and 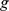 the glycosylation rates. These linear reaction rates represent the initial slope of the Michaelis Menten curve of the enzymes, that is, 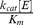. A linear degradation term is present in the equations of the modified flavonoids (eqs. (4), (7), (10), (13)).

Parameter values were iteratively changed to find the best fit to the data. The relative distance between the measured metabolite concentrations (Figure 2) and the model results, measured by the sum of relative errors, was optimized. Additionally, a weighted penalty was added for intermediates that accumulate to values above the experimental threshold (taken as 2 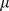
mol kg^−1^).

The optimization started with a MATLAB global genetic algorithm (ga) followed by the local search algorithm FMINCON.

#### Model 1

Enzyme concentrations were assumed constant during the experiment, and were allowed to vary freely between tissues. The 22 catalytic rates (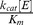, for example 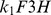 and 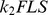 in eq. 2–13) are estimated separately for cotyledons and hypocotyls, resulting in a total of 44 parameters to fit the data.

#### Model 2

Instead of assuming constant enzyme concentrations, we used curves fitted to enzymatic expression data (Figure 4). The curves were fitted with a Piecewise Cubic Hermite Interpolating Polynomial (PCHIP), and transcript abundance was extracted from these curves during the solving of the ODEs. Because a single set of enzymes functions in both tissues of the seedling, the enzymatic rates (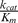), should be equal in both tissues. Only the reduction rates were allowed to vary between tissues. Therefore, the model with 22 pars was first fitted to the cotyledons data and then applied to the hypocotyls data. Only the 4 reduction rates were allowed to vary between the tissues. Note, however, that all 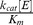 values vary between tissues according to the measured differential transcript abundance.

## Supporting Information

Figure S1 **Comparison of gene expression measurements in a quantitative RT-PCR platform (left panels) and in a micro-array platform.** Cotyledon and hypocotyl tissues on different days are represented. The values indicate gene expression relative to L33 (left) and micro-array signal intensity (right).
(TIFF)

Table S1 Candidate genes identified by their annotation in ITAG2.30 were scored for expression intensity on the micro-array and by sequence similarity to Petunia x hybrida genes that have been experimentally implicated in flavonoid metabolism.
(XLSX)
