# Supplementary figures and images for: The Flavonoid Pathway in Tomato Seedlings: Transcript Abundance and the Modeling of Metabolite Dynamics
Source: PLoS One. 2013 Jul 26;8(7):e68960. doi: 10.1371/journal.pone.0068960 (PMC3724892; doi:10.1371/journal.pone.0068960)

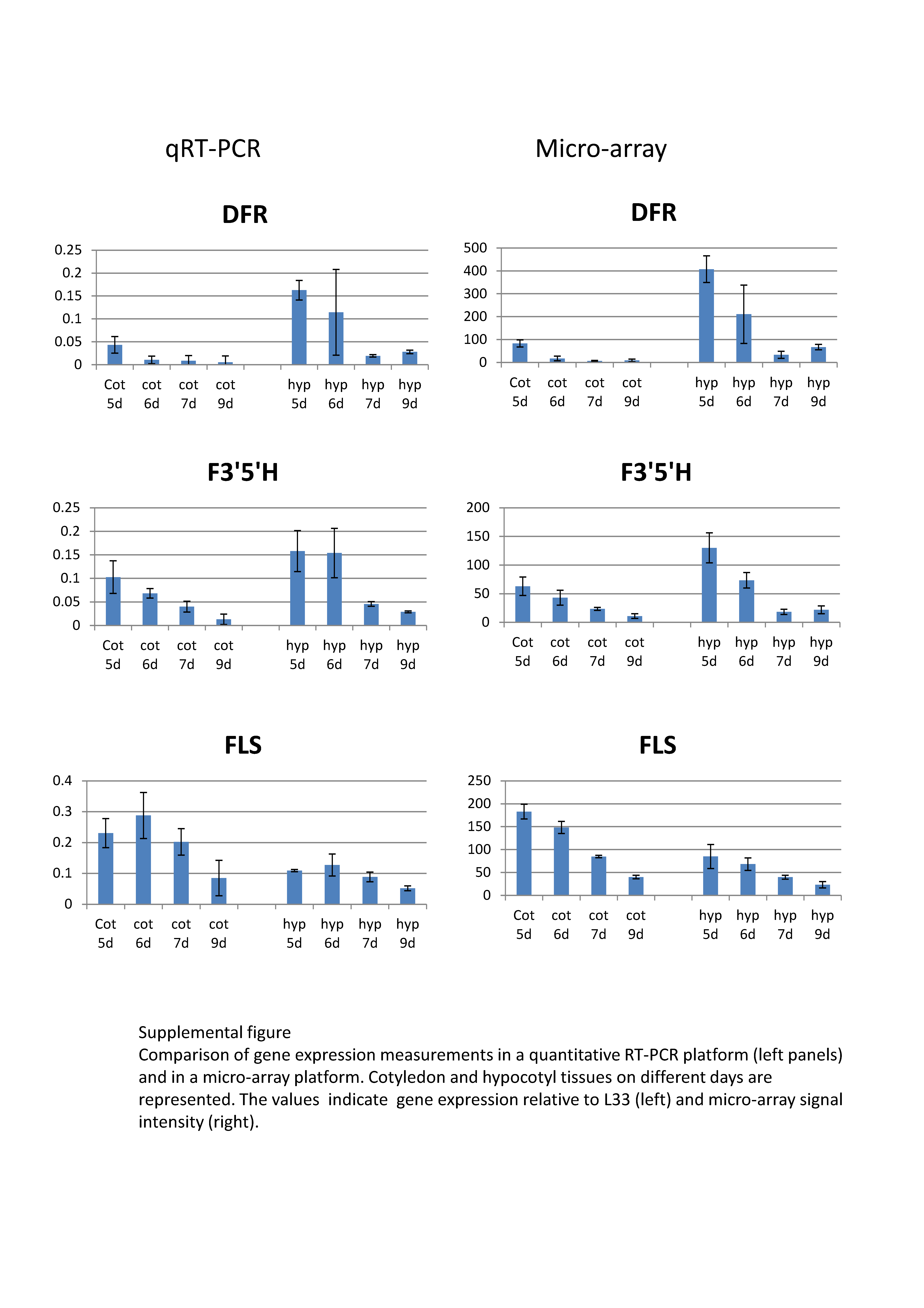

Supplement: Figure S1 — Comparison of gene expression measurements in a quantitative RT-PCR platform (left panels) and in a micro-array platform. Cotyledon and hypocotyl tissues on different days are represented. The values indicate gene expression relative to L33 (left) and micro-array signal intensity (right). (TIFF) [file pone.0068960.s001.tiff]
